# The right kind of rarefaction: Coronary microvascular remodeling in right ventricle failure

**DOI:** 10.1016/j.jhlto.2025.100459

**Published:** 2025-12-02

**Authors:** Cyrus Vahdatpour, Katharine Clapham, Steven M. Kawut, Kirk Jones, John J. Ryan, Danielle Petty, Shannon Talbot, Kimberly Dumoff, Ellen C. Keeley, Priti Lal, Andrew J. Bryant, Alex M. Parker, Jeremy A. Mazurek, Leonid Mirson, Andrew Murphy, Andrew Baird, Megan Schwietert, Alissa Schurr, Andrew Stein, Scott M. Hansen, Zhining Ou, Angela P. Presson, Dylan Miller

**Affiliations:** a Division of Pulmonary and Critical Care Medicine, Baylor College of Medicine, Houston, TX; b Division of Cardiovascular Medicine, University of Utah, Salt Lake City, UT; c Division of Pulmonary, Allergy, and Critical Care Division, Perelman School of Medicine at the University of Pennsylvania, Philadelphia, PA; d Division of Pulmonary and Critical Care Medicine, University of Florida, Gainesville, FL; e Division of Pathology, University of Florida, Gainesville, FL; f Division of Pathology, Perelman School of Medicine at the University of Pennsylvania, Philadelphia, PA; g Division of Cardiology, University of Florida, Gainesville, FL; h Division of Cardiology, Perelman School of Medicine at the University of Pennsylvania, Philadelphia, PA; i Division of Medicine, Pennsylvania Hospital, Philadelphia, PA; j Division of Pathology, Thomas Jefferson University, Philadelphia, PA; k Division of Cardiology, University of Alabama, Birmingham, AL; l College of Life Sciences, Bringham Young University, Provo, UT; m Division of Epidemiology, Department of Internal Medicine, School of Medicine, University of Utah, Salt Lake City, UT; n Division of Pathology, University of Utah, Salt Lake City, UT

**Keywords:** Pulmonary hypertension, Right ventricular failure, Coronary microvascular dysfunction, Heart transplantation, Digital pathology, Capillary rarefaction

## Abstract

**Background:**

Right ventricular failure (RVF) is the primary determinant of outcomes in pulmonary hypertension (PH). Coronary microvascular dysfunction (CMD), defined by capillary rarefaction and endothelial dysfunction, may contribute to RVF but remains poorly characterized. CMD, defined by capillary rarefaction and endothelial dysfunction, may contribute to RVF through impaired myocardial oxygen delivery and fibrotic remodeling.

**Objectives:**

To characterize right ventricle (RV) CMD and myocardial fibrosis in explanted human hearts and examine associations with echocardiographic and hemodynamic indices of RVF across PH subtypes.

**Methods:**

We retrospectively analyzed 57 adult patients who underwent orthotopic heart transplantation at 3 institutions (2023-2024). Explanted hearts were classified by PH subtype: combined pre-/post-capillary PH (CpcPH, *n* = 24), isolated post-capillary PH (IpcPH, *n* = 22), and no PH (*n* = 11). Digital pathology quantified RV and left ventricle (LV) capillary density and interstitial fibrosis across epicardial, mid-wall, and endocardial regions. Associations with echocardiographic measures and hemodynamic parameters were assessed.

**Results:**

A total of 57 hearts (70% male, median age 52 years) were analyzed. Median time from listing to transplantation was 1.6 months (IQR: 0.7-6.1). Mean RV capillary density was 653 ± 204 microvessels/mm^2^ and correlated significantly with TAPSE (ρ_s_ = 0.49, *p* < 0.001) and tricuspid annular plane systolic excursion to systolic pulmonary artery pressure (TAPSE/sPAP) ratio (ρ_s_ = 0.33, *p* = 0.037). Compared to patients without PH, patterns of reduced mid-wall capillary density in PH subtypes were observed (CpcPH: β = −228 microvessels/mm^2^, *p* = 0.031; IpcPH: β = −238 microvessels/mm^2^, *p* = 0.043). Diabetes mellitus was associated with reduced LV sub-endocardial capillary density (β = −207, *p* = 0.006). Unadjusted analysis showed higher RV fibrosis in patients without PH (*p* = 0.024); however, after adjusting for clinical confounders, including heart failure etiology, this difference was not significant, highlighting the heterogenous nature of fibrosis in end-stage heart failure.

**Conclusions:**

Capillary rarefaction is a measurable histopathologic feature in explanted RV tissue that correlates with functional indices of RV performance. Our proof-of-concept findings suggest CMD may contribute to RV systolic dysfunction independent of PH subtype.

## Background

### Pulmonary hypertension in advanced heart failure

Pulmonary hypertension (PH) is a common complication of advanced heart failure and is associated with poor prognosis. The 2022 European Society of Cardiology (ESC)/European Respiratory Society Guidelines define PH as elevated mean pulmonary artery (PA) pressure (≥20mmHg) and stratify it into pre-, post-, and combined pre-/post-capillary subtypes with implications for therapy and heart transplant candidacy.^1^ Significant elevation in pulmonary vascular resistance (PVR) is a contraindication to heart transplant, increasing the risk of primary graft failure and early mortality.^2^ Right Ventricle (RV)– PA uncoupling, defined as the progressive loss of contractile compensation for afterload, has emerged as a key feature of decompensated PH and an independent predictor of pre-transplant outcomes.^3^, ^4^, ^5^, ^6^ Noninvasive surrogates of RV–PA coupling, such as the ratio of tricuspid annular plane systolic excursion to systolic pulmonary artery pressure (TAPSE:sPAP ratio), are increasingly used in clinical risk stratification and have been associated with RV contractile reserve, ventricular remodeling, and survival.^4^, ^7^

### Coronary microvascular dysfunction as a mechanism in pulmonary hypertension

While RV afterload has historically dominated the paradigm of right ventricular failure in PH, growing evidence implicates coronary microvascular dysfunction (CMD) as an underrecognized contributor to RV decompensation.^8^, ^9^, ^10^ CMD, defined as endothelial dysfunction and corresponding ischemia, may impair RV oxygen delivery through capillary rarefaction and subsequent myocardial fibrosis.^11^ Preclinical models suggest that microvascular rarefaction precede overt vascular remodeling, leading to RV failure in PH.^11^, ^12^ To our knowledge, RV CMD has not been previously characterized histologically in patients with Group 2 PH, and its association with RV-PA uncoupling remains unknown.

#### Coronary microvascular dysfunction in heart failure with preserved ejection fraction and cardiac allograft vasculopathy—mechanistic overlap with right ventricle failure

CMD plays a central role in the pathophysiology of heart failure with preserved ejection fraction (HFpEF) and is well described in cardiac allograft vasculopathy (CAV), both of which feature impaired capillary function, diastolic dysfunction, and fibrosis of the left ventricle (LV).^13^, ^14^ In HFpEF, CMD is linked to impaired perfusion reserve, inflammation, and fibrosis, contributing to exercise intolerance and chamber stiffening.^15^ In explanted CAV hearts, reduced capillary density correlates with diastolic dysfunction and clinical symptoms.^14^, ^16^ Despite these mechanistic overlaps, CMD has not been systematically explored in PH or in explanted RV tissue—where the implications for myocardial reserve and RV-PA coupling may be profound for those undergoing transplant evaluation.

### Histologic assessment of coronary microvascular dysfunction and fibrosis

CD31 immunohistochemistry is a validated method to quantify myocardial capillary density.^13^, ^14^, ^16^ Trichrome staining is the standard method for evaluating myocardial fibrosis, particularly interstitial collagen deposition. Recent advances in digital pathology, including supervised machine-learning models, now allow for reproducible quantification of histologic features such as vascular rarefaction and fibrosis from formalin-fixed, paraffin-embedded tissue.^17^ These techniques enhance the ability to link histopathologic remodeling to clinical metrics like invasive and noninvasive hemodynamics and may identify histologic correlates of RV-PA uncoupling.

### Hypothesis and study aims

We hypothesize that coronary microvascular rarefaction and interstitial myocardial fibrosis are associated with hemodynamic sequela in patients with and without PH, and that these histopathologic changes define distinct myocardial phenotypes. Our study aimed to (1) quantify RV capillary density and fibrosis in explanted human hearts, (2) study the association with echocardiographic and hemodynamic indices of RV-PA uncoupling, and (3) identify histopathologic patterns associated with PH subtypes that may underlie RV failure phenotypes in advanced heart failure.

## Methodology

### Study oversight and IRB approval

This study was approved by a multi-institutional review board protocol.

### Study subjects

We retrospectively identified 57 adult patients who underwent orthotopic heart transplantation from 2023 to 2024 at the University of Florida, University of Pennsylvania and University of Utah. Explanted heart specimens from these patients were collected for histopathologic analysis, and clinical data were abstracted from the electronic medical records using standardized data abstraction forms. All patients had right and left ventricular tissue samples available for analysis and echocardiographic and right heart catheterization data. Timing from listing to transplantation was recorded as a proxy for the interval between functional assessment and tissue collection, recognizing that echocardiographic and hemodynamic evaluations are typically performed within 3-6 months prior to listing per institutional protocols. PH hemodynamic subtype distinctions were based on the ESC/ERS guidelines.^1^

### Data abstraction

Demographics, PH subtype, hemodynamic variables, and echocardiographic metrics were recorded. Data were entered into REDCap using a centralized data dictionary shared across sites. Data abstraction was blinded to the histological characteristic acquisition.

#### Histopathology sample preparation

As part of consented research protocols at each institution, full-thickness tissue sections were obtained from each ventricle at the time of explant during cardiac transplantation. The tissues were fixed in 10% neutral buffered formalin and processed to paraffin blocks. Estimated average time in formalin before processing was 12 to 72 hours. Sections from each block (right and LV) were stained using standard hematoxylin-eosin, Masson trichrome, and CD31 immunoperoxidase stains at each institution. The slides were digitally scanned using a Hamamatsu S360 Digital Slide Scanner and analyzed using standardized automated algorithms, QuPath open source software, to ensure objective, reproducible quantification across all centers.^18^

#### Digital image algorithm and analysis

**Figure 1:**
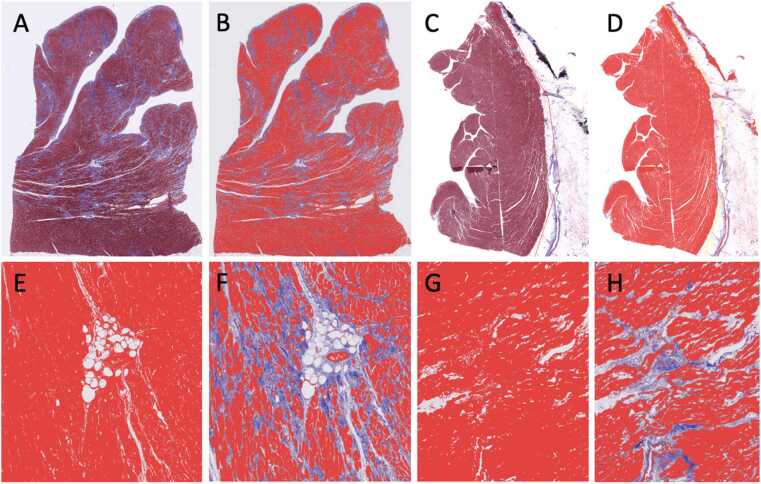
*Digital pathology quantitation of fibrosis.* Low-power view of a full-thickness left ventricle (A) and right ventricle (C) sections stained with Masson trichome. (B) and (D) Digital quantitation of myocyte area (red channel); pixels meeting the myocyte (red) threshold artificially appear bright red in this markup image (note the pale blue stained areas are not part of the detection area). (E) and (G) Digital quantitation of the total tissue area; only white (background) pixels are excluded, such as the central fatty tissue and large vessel lumens. (F) and (H) Digital quantitation of the myocyte areas (red channel) of the same tissue region as shown in (E) and (G); the total area of blue collagen (fibrosis) is calculated indirectly by subtracting the myocyte area from the total tissue area.

**Figure 2:**
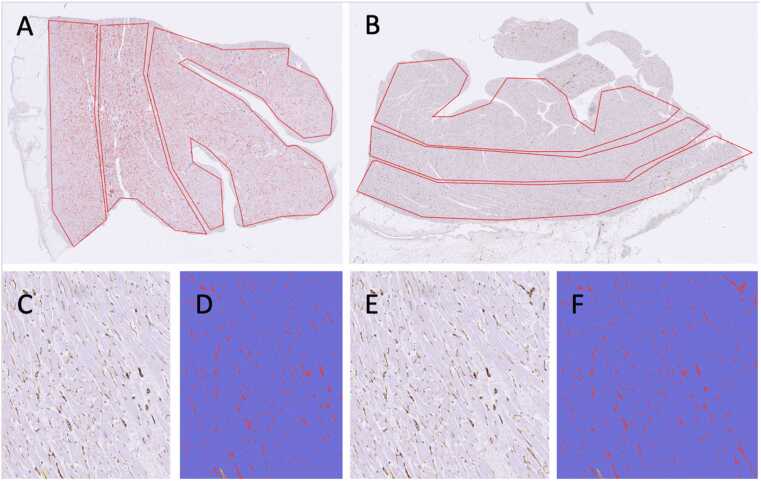
*Digital pathology quantitation of microvessel density.* (A) and (B) Low-power view of a full-thickness section of the left ventricle stained with CD31 immunohistochemistry showing manual annotations segmenting the wall into 3 regions (subendocardial, mid-wall, and subepicardial). Red pseudocolor labeling of structures identified by the digital algorithm to be microvessels can also be seen from this magnification. (C) and (E) High-power view of the native CD31-stained slide image showing DAB labeling of microvessels. (D) and (F) Digital object classification applied to objects meeting the DAB threshold of the quantitation algorithm, confirming sensitive and specific detection of microvessels. DAB, diamino benzidin.

Digital image analysis using QuPath software eliminated operator-dependent variability through automated, algorithm-based quantification. Detection thresholds were standardized across institutions using representative training images, ensuring objective and reproducible measurements of capillary density and fibrosis. QuPath was utilized to tune an algorithm for quantifying the amount of tissue fibrosis using the trichrome stain and microvessel density (# microvessels per unit area) using the CD31 immunostain. For fibrosis quantitation, detection thresholds were set manually using several representative image files as a training set. One threshold was set for detection of the entire tissue area (excluding any gaps, “air,” or empty space between small tears in the tissue section, etc. A second threshold was set for detection in the red channel of myocyte staining. Using these 2 thresholds, the amount of fibrosis (collagen area) was determined indirectly (total tissue area–myocyte area = fibrosis (collagen area) (Figure 1). This was found to be a more accurate and consistent means of quantification compared to directly measuring the collagen content (blue channel), due to variation in the blue stain intensity from slide to slide and institution to institution. For microvessel quantitation, a detection threshold was set to segment the diamino benzidine signal from the background hematoxylin staining. Using that pixel classifier threshold, the Create Objects tool was used to automatically count each vessel with the minimum object size set to 20 μm^2^, minimum hole size 200 μm^2^, and “Split Objects” enabled. Manual visual checking of several representative CD31 slides confirmed accurate and specific automatic detection of microvessels. All trichrome and CD31 slides were analyzed using these same settings in QuPath. For the trichrome stains, the entire section was selected. For the CD31 stains, separate regions for analysis were manually drawn, segmenting the subepicardial, mid wall, and subendocardial regions (Figure 2).

### Echocardiography and hemodynamic assessment

Transthoracic echocardiogram reports obtained at the time closest to transplant listing were reviewed. The TAPSE/sPAP ratio was calculated as a surrogate for RV-PA coupling. Right heart catheterization data were collected from pre-transplant hemodynamic evaluations at the time closest to transplant listing.

### Statistical analysis

Continuous variables were expressed as mean ± standard deviation and median (interquartile range, IQR). Categorical variables were presented as frequencies and percentages. Comparisons among PH subtypes were performed using Kruskal-Wallis tests for continuous variables and χ² or Fisher’s exact tests for categorical variables. Spearman’s rank correlation coefficients (ρ_s_) between histologic features and echocardiographic or hemodynamic parameters were assessed. Correlation and regression analysis were primary presented without adjustment for center and secondarily (online) adjusted for center. The corresponding 95% confidence intervals (CIs) were calculated using the “spearman.ci” function from R package “RVAideMemoire,” and 1,000 bootstrapping sample using “boot” function from R package “boot.” The strength of relationship can be interpreted as very weak (0-0.19), weak (0.20-0.39), moderate (0.40-0.59), strong (0.60-0.79), and very strong (0.80-1).^19^ Univariable and multivariate regression models were used to identify independent associations between RV capillary density, fibrosis, and RV-PA coupling indices (TAPSE/sPAP, PAPi). Similar independent associations were performed for LV measurements. We adjusted for age, chronic kidney disease (CKD), and diabetes mellitus (DM) in multivariable models a priori. We repeated the multivariable regression analysis with additional adjustment for institutions and present in supplements. We reported mean LV/RV capillary density as the primary outcome, the rest of the 3 regions (epicardial, mid-wall, and subendocardial) are secondary outcomes. Statistical significance was assessed at the 0.05 level, and no adjustment for multiple comparisons was performed. Statistical analyses were implemented using R v. 4.4.3.^20^

## Results

### Clinical characteristics

A total of 57 hearts from patients undergoing orthotopic heart transplantation were included. The cohort included individuals with and without PH, including isolated post-capillary PH (IpcPH) and combined pre- and post-capillary PH (CpcPH). Baseline demographics and comorbidities are summarized in Table 1. There were no significant differences in age, gender, or heart failure phenotype across PH subtypes. Inotrope use at the time of transplant was more frequent in patients with CpcPH and IpcPH compared to those without PH (88%, 86%, vs 45%, *p* = 0.019). CKD was more prevalent among patients with PH compared to those without (*p* = 0.047), with the greatest prevalence observed in IpcPH (64%). Table S1 summarizes demographics and co-morbidities stratified by institute.

**Table 1:** Summary of Demographics and Clinical Characteristics Stratified by PH Subtypes

Variable	Total (*N* = 57)	Combined pre and post capillary PH (*N* = 24)	Isolated post capillary PH (*N* = 22)	No PH (*N* = 11)	*p*-value
*Gender -* Female	17(30%)	9(38%)	5(23%)	3(27%)	0.56^f^
Male	40(70%)	15(62%)	17(77%)	8(73%)	-
*Age -* Mean (SD)	52(14)	50 (15)	51 (13)	57 (10)	0.27^k^
Median (IQR)	56(45,62)	54 (43, 60)	52 (44,61)	62 (53, 64)	-
Range	(21,70)	(21, 69)	(24, 70)	(37, 68)	-
*Race -* American Indian	1(1.8%)	1(4.2%)	0(0%)	0(0%)	0.46^f^
Asian	1(1.8%)	0(0%)	1(4.5%)	0(0%)	-
Black	7(12%)	5(21%)	2(9.1%)	0(0%)	-
Hispanic/Latino	10(18%)	5(21%)	4(18%)	1(9.1%)	-
White	38(67%)	13(54%)	15(68%)	10(91%)	-
*BMI -* 18.5-24.9	17(30%)	6(25%)	5(23%)	6(55%)	0.54^f^
25-29.9	24(42%)	12(50%)	10(45%)	2(18%)	-
30-34.9	10(18%)	4(17%)	4(18%)	2(18%)	-
35 or higher	6(11%)	2(8.3%)	3(14%)	1(9.1%)	-
*Etiology of heart failure -* Arrhythmia	5(8.8%)	1(4.2%)	2(9.1%)	2(18%)	0.67^f^
Congenital	9(16%)	3(12%)	4(18%)	2(18%)	-
Ischemic	11(19%)	4(17%)	4(18%)	3(27%)	-
Myocarditis	3(5.3%)	2(8.3%)	0(0%)	1(9.1%)	-
Other	28(49%)	13(54%)	12(55%)	3(27%)	-
Valvular	1(1.8%)	1(4.2%)	0(0%)	0(0%)	-
*Pacemaker implanted -* No	34(60%)	16(67%)	13(59%)	5(45%)	0.50^f^
Yes	23(40%)	8(33%)	9(41%)	6(55%)	-
*ICD implanted -* No	14(25%)	9(38%)	4(18%)	1(9.1%)	0.14^f^
Yes	43(75%)	15(62%)	18(82%)	10(91%)	-
*Hypertension -* No	28(49%)	13(54%)	11(50%)	4(36%)	0.62^c^
Yes	29(51%)	11(46%)	11(50%)	7(64%)	-
*Diabetes mellitus -* No	42(74%)	18(75%)	15(68%)	9(82%)	0.79^f^
Yes	15(26%)	6(25%)	7(32%)	2(18%)	-
*Latest HbA1c -* Mean (SD)	5.8 (0.96)	5.8 (0.91)	5.9 (1.1)	5.7 (0.59)	0.66^k^
Median (IQR)	5.7 (5.3,6.3)	5.7 (5.3,6.1)	5.8 (5.4,6.4)	5.5 (5.4, 5.7)	-
Range	(3.5,8.6)	(3.5, 8.0)	(4.0,8.6)	(4.9, 7.0)	-
*Chronic kidney disease -* No	30(53%)	13(54%)	8(36%)	9(82%)	0.047^c^
Yes	27(47%)	11(46%)	14(64%)	2(18%)	-
*Hyperlipidemia -* No	28(49%)	12(50%)	9(41%)	7(64%)	0.47^c^
Yes	29(51%)	12(50%)	13(59%)	4(36%)	-
*Peripheral vascular disease -* No	51(89%)	21(88%)	20(91%)	10(91%)	1.00^f^
Yes	6(11%)	3(12%)	2(9.1%)	1(9.1%)	-
*OSA/OHVS -* No	43(75%)	20(83%)	13(59%)	10(91%)	0.10^f^
Yes	14(25%)	4(17%)	9(41%)	1(9.1%)	-
*Use of inotrope prior to transplant -* No	12(21%)	3(12%)	3(14%)	6(55%)	0.019^f^
Yes	45(79%)	21(88%)	19(86%)	5(45%)	-
*Use of vasopressor prior to transplant -* No	38(67%)	13(54%)	16(73%)	9(82%)	0.23^f^
Yes	19(33%)	11(46%)	6(27%)	2(18%)	-
*Use of MCS inotrope -* Checked	21(37%)	9(38%)	6(27%)	6(55%)	0.33^f^
Unchecked	36(63%)	15(62%)	16(73%)	5(45%)	-
*Time on waiting list -* >1 year	6(11%)	2(8.3%)	2(9.1%)	2(18%)	0.54^f^
1-3 months	13(23%)	4(17%)	8(36%)	1(9.1%)	-
1 week to 1 month	15(26%)	10(42%)	3(14%)	2(18%)	-
3-6 months	11(19%)	4(17%)	4(18%)	3(27%)	-
6-12 months	7(12%)	2(8.3%)	3(14%)	2(18%)	-
Less than 1 week	5(8.8%)	2(8.3%)	2(9.1%)	1(9.1%)	-
*Waitlist decompensations -* more than 3 (transplant from outpatient)	1(1.8%)	1(4.2%)	0(0%)	0(0%)	0.09^f^
None (came in from outpatient)	13(23%)	4(17%)	5(23%)	4(36%)	-
One (came in from outpatient)	7(12%)	5(21%)	0(0%)	2(18%)	-
One (lead to transplant)	29(51%)	13(54%)	12(55%)	4(36%)	-
Three (final led to transplant)	1(1.8%)	1(4.2%)	0(0%)	0(0%)	-
Two (final lead to transplant)	5(8.8%)	0(0%)	4(18%)	1(9.1%)	-
Two (transplant from outpatient)	1(1.8%)	0(0%)	1(4.5%)	0(0%)	-

Abbreviations: BMI, body mass index; ICD, implantable cardioverter-defibrillator; IQR, interquartile range; MCS, mechanical circulatory support; OHVS, obesity hypoventilation syndrome; OSA, obstructive sleep apnea.

Missing values: Latest HbA1c = 0 (0%)/0 (0%)/1 (9.1%).

f Fisher's exact test.

k Kruskal-Wallis test.

c Chi-squared test.

The median time from listing to transplantation was 1.6 months (IQR:0.7-6.1 months). Most patients (73.6%) underwent transplantation within 6 months of listing, with 42 patients (73.6%) having ≤6 months and only seven patients (12.3%) waiting >12 months.

### Histopathology characteristics

Quantitative analysis of RV and LV tissue revealed heterogeneous distributions of capillary density and fibrosis between PH subtypes (Table 2). CD31 immunostaining showed variability in microvascular rarefaction amongst cohorts, and trichrome staining identified interstitial fibrosis across all groups. Patients with PH did not uniformly demonstrate greater fibrosis than those without PH. Figure S1 displays boxplots of capillary density, percent fibrosis, and collagen:myocyte ratio across PH subtypes within LV/RV measurement. Across PH subtypes, a higher RV fibrosis percentage was seen in patients without PH compared to those with PH (*p* = 0.024, Table 2).

**Table 2:** Histopathology Characteristics Stratified by PH Subtypes

Variable	Total (*N* = 57)	Combined pre and post capillary PH (*N* = 24)	Isolated post capillary PH (*N* = 22)	No PH (*N* = 11)	*p*-value
*RV capillary density mean* - Mean (SD)	653 (204)	635 (198)	656 (206)	688 (227)	0.77^k^
Median (IQR)	653 (549, 761)	635 (555, 750)	643 (487, 832)	699 (601, 760)	-
Range	(57, 1,067)	(57, 1,038)	(196, 968)	(254, 1,067)	-
*RV capillary density sub epicardium* - Mean (SD)	663 (258)	620 (275)	664 (265)	782 (173)	0.31^k^
Median (IQR)	636 (471, 881)	603 (445, 736)	611 (475, 906)	864 (675, 893)	-
Range	(40, 1,236)	(40, 1,236)	(159, 1,017)	(494, 980)	-
*RV capillary density mid layer* - Mean (SD)	656 (225)	609 (216)	651 (240)	799 (174)	0.15^k^
Median (IQR)	658 (522, 802)	636 (459, 726)	616 (522, 837)	797 (711, 831)	-
Range	(71, 1,135)	(71, 1,032)	(193, 1,036)	(577, 1,135)	-
*RV capillary density sub endocardium* - Mean (SD)	745 (272)	701 (267)	769 (244)	811 (348)	0.62^k^
Median (IQR)	765 (601, 913)	705 (595, 867)	812 (611, 960)	827 (656, 932)	-
Range	(59, 1,417)	(59, 1,113)	(235, 1,055)	(254, 1,417)	-
*RV % fibrosis -* Mean (SD)	0.30 (0.10)	0.28 (0.08)	0.28 (0.11)	0.38 (0.11)	0.024^k^
Median (IQR)	0.27 (0.24, 0.38)	0.26 (0.23, 0.31)	0.26 (0.22, 0.34)	0.39 (0.32, 0.40)	-
Range	(0.07, 0.62)	(0.12, 0.42)	(0.07, 0.53)	(0.22, 0.62)	-
*RV collagen: myocyte ratio -* Mean (SD)	0.49 (0.31)	0.42 (0.18)	0.49 (0.37)	0.66 (0.35)	0.06^k^
Median (IQR)	0.37 (0.31, 0.63)	0.35 (0.31, 0.56)	0.35 (0.29, 0.57)	0.64 (0.48, 0.66)	-
Range	(0.08, 1.7)	(0.14, 0.74)	(0.08, 1.7)	(0.28, 1.6)	-
*LV capillary density mean -* Mean (SD)	618 (209)	604 (231)	598 (164)	684 (237)	0.55^k^
Median (IQR)	608 (516, 708)	603 (521, 682)	562 (518, 718)	684 (516, 877)	-
Range	(42, 1,289)	(42, 1,289)	(326, 942)	(258, 1,020)	-
*LV capillary density sub epicardium -* Mean (SD)	647 (268)	579 (267)	695 (226)	716 (351)	0.29^k^
Median (IQR)	630 (470, 793)	547 (454, 647)	700 (548, 857)	657 (507, 882)	-
Range	(9.3, 1,313)	(9.3, 1,305)	(332, 1,182)	(265, 1,313)	-
*LV capillary density mid layer -* Mean (SD)	545 (240)	537 (236)	511 (199)	641 (337)	0.81^k^
Median (IQR)	471 (391, 668)	560 (416, 654)	465 (381, 607)	418 (388, 890)	-
Range	(44, 1,175)	(44, 1,082)	(253, 922)	(338, 1,175)	-
*LV capillary density sub endocardium -* Mean (SD)	592 (225)	564 (230)	579 (230)	695 (202)	0.34^k^
Median (IQR)	586 (468, 736)	594 (471, 624)	562 (436, 717)	638 (582, 849)	-
Range	(74, 1,133)	(74, 1,007)	(266, 1,133)	(394, 971)	-
*LV % fibrosis -* Mean (SD)	0.37 (0.13)	0.36 (0.13)	0.39 (0.14)	0.35 (0.10)	0.72^k^
Median (IQR)	0.37 (0.27, 0.46)	0.37 (0.27, 0.45)	0.38 (0.28, 0.50)	0.33 (0.27, 0.44)	-
Range	(0.16, 0.70)	(0.16, 0.70)	(0.17, 0.61)	(0.23, 0.51)	-
*LV collagen: myocyte ratio -* Mean (SD)	0.68 (0.42)	0.67 (0.45)	0.74 (0.47)	0.56 (0.26)	0.71^k^
Median (IQR)	0.58 (0.38, 0.85)	0.61 (0.38, 0.82)	0.62 (0.40, 1.1)	0.48 (0.37, 0.80)	-
Range	(0.19, 2.3)	(0.19, 2.3)	(0.21, 1.7)	(0.29, 1.0)	-

Abbreviations: IQR, interquartile range; RV, right ventricle; LV, left ventricle; PH, pulmonary hypertension.

Missing values: RV capillary density mean = 0 (0%)/2 (9.1%)/0 (0%), RV capillary density sub epicardium = 4 (17%)/7 (32%)/4 (36%), RV capillary density mid layer = 4 (17%)/7 (32%)/4 (36%), RV capillary density sub endocardium = 4 (17%)/7 (32%)/3 (27%), RV % fibrosis = 0 (0%)/2 (9.1%)/0 (0%), RV collagen: myocyte ratio = 1 (4.2%)/1 (4.5%)/0 (0%), LV capillary density mean = 0 (0%)/2 (9.1%)/0 (0%), LV capillary density sub epicardium = 6 (25%)/6 (27%)/4 (36%), LV capillary density mid layer = 6 (25%)/6 (27%)/4 (36%), LV capillary density sub endocardium = 6 (25%)/6 (27%)/4 (36%), LV % fibrosis = 0 (0%)/2 (9.1%)/0 (0%), LV collagen: myocyte ratio = 1 (4.2%)/1 (4.5%)/0 (0%).

k Kruskal-Wallis test.

### Histopathology correlates of cardiac function

**Figure 3:**
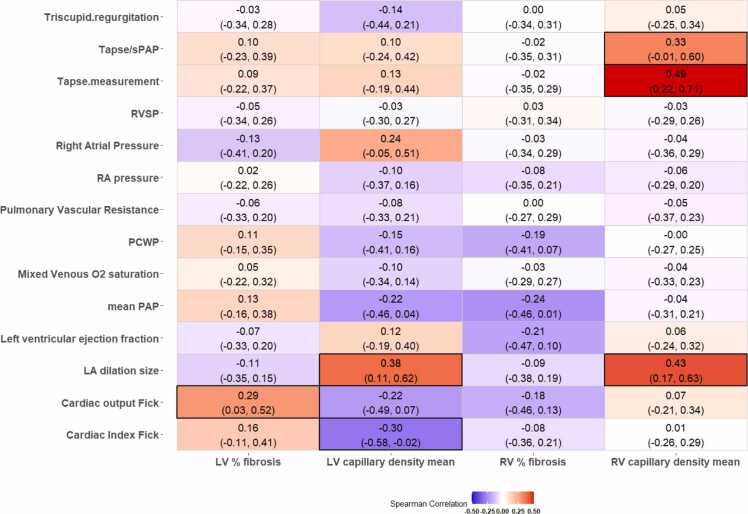
*Heatmap for unadjusted Spearman’s rank correlation coefficients and 95% confidence intervals. Black borders indicate statistical significance.* LA, left atrium; PAP, pulmonary artery pressure; PCWP, pulmonary capillary wedge pressure; RA, right atrium; RVSP, right ventricular systolic pressure; sPAP, systolic pulmonary artery pressure; TAPSE, tricuspid annular plane systolic excursion.

**Figure 4:**
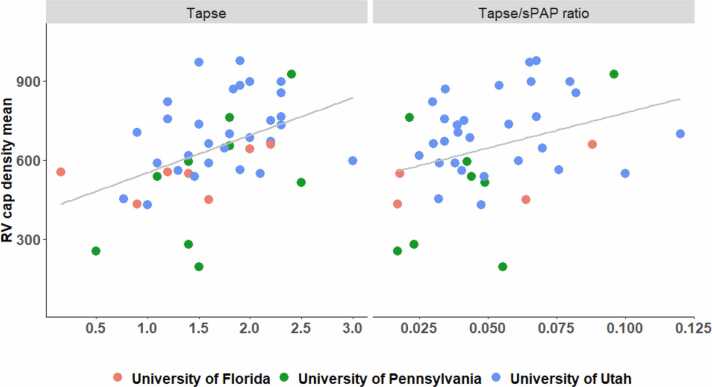
*Scatterplot of RV capillary density mean versus TAPSE measurement.* Legend: Scatterplot shows the correlation between mean RV capillary density and TAPSE across PH subtypes. Each point represents one patient; colors indicate the institution of each patient. The regression line demonstrates the positive correlation from Figure 3. RV, right ventricle; TAPSE, tricuspid annular plane systolic excursion; PH, pulmonary hypertension.

We assessed unadjusted correlations between histologic features and cardiac functional measures in a heatmap for all correlations in Figure 3. Table S3 represents these correlations adjusted by center. Mean RV capillary density increased modestly in association with TAPSE (ρ_s_ = 0.49, 95% CI: 0.23-0.71); *p* < 0.001) and weakly correlated with TAPSE/sPAP ratio (ρ_s_ = 0.33, 95% CI: 0.002-0.58); *p* = 0.037), visualized in Figure 4.

Left atrium diameter demonstrated positive correlation with both mean RV capillary density (ρ_s_ = 0.43, 95% CI: 0.17-0.63); *p* = 0.002) and mean LV capillary density (ρ_s_ = 0.38, CI: 0.09-0.61; *p* = 0.006). LV capillary density was inversely correlated with cardiac index (ρ_s_ = −0.30, 95% CI:−0.55 to −0.04; *p* = 0.025), while similar relationship was not seen between RV capillary density and CI. RV fibrosis was not significantly associated with any hemodynamic or echocardiographic measures. Percent fibrosis of the LV positively correlated with cardiac output (ρ_s_ 0.29, 95% CI:0.02-0.52); *p* = 0.036).

### Association between fibrosis and microvascular rarefaction

Pairwise partial Spearman correlation analysis was performed to evaluate the internal consistency among regional capillary densities and their relationship to fibrosis, as shown in Table S4 (unadjusted for center) and summarized in Table S5 (adjusted for center).

### Univariate analysis of histopathologic predictors

The result of univariate linear regression assessed associations between clinical variables and capillary density without adjustment for center were shown in Table 3. In the RV, increasing age (per year) was significantly associated with 8.17 microvessels/mm^2^ higher sub-epicardial capillary density (β = 8.17, 95% CI 2.35-13.99, *p* = 0.009). No additional clinical predictors—including CKD, DM, or PH subtype—were significantly associated with RV capillary density. In the LV, however, DM was significantly associated with 151.78 microvessels/mm^2^ lower sub-endocardial capillary density (β = −151.78, 95% CI: −294.07 to −9.50, *p* = 0.043). No other clinical predictors were significantly associated with regional or mean LV capillary density. Table S6 is the same “univariate” analysis but controlled for center.

**Table 3:** Univariable Linear Regression Models

	RV capillary density mean			RV capillary density sub epicardium		
Variable	Coefficient (95% CI)	*p*-value	*N*	Coefficient (95% CI)	*p*-value	*N*
*Type of explant -* combined	−52.89 (−200.50,94.73)	0.49	55	−162.43 (−384.47,59.62)	0.16	42
Isolated	−31.85 (−184.03,120.33)	0.68	55	−118.05 (−349.49,113.40)	0.32	42
Age	3.63 (−0.19,7.45)	0.07	55	8.17 (2.35,13.99)	0.009	42
Chronic kidney disease	92.21 (−13.90,198.32)	0.09	55	83.97 (−72.14,240.08)	0.30	42
Diabetes mellitus	56.67 (−64.53,177.88)	0.36	55	95.12 (−77.35,267.59)	0.29	42

	RV capillary density mid layer			RV capillary density sub endocardium		
Variable	Coefficient (95% CI)	*p*-value	*N*	Coefficient (95% CI)	*p*-value	*N*
*Type of explant -* Combined	−190.35 (−379.33,−1.37)	0.06	42	−109.14 (−335.02,116.73)	0.35	43
Isolated	−148.13 (−345.11,48.85)	0.15	42	−41.33 (−277.71,195.06)	0.73	43
Age	2.97 (−2.47,8.42)	0.29	42	2.07 (−4.55,8.69)	0.54	43
Chronic kidney disease	81.21 (−54.17,216.59)	0.25	42	98.37 (−63.72,260.46)	0.24	43
Diabetes mellitus	85.12 (−64.82,235.05)	0.27	42	43.93 (−139.30,227.17)	0.64	43

	LV capillary density mean			LV capillary density sub epicardium		
Variable	Coefficient (95% CI)	*p*-value	*N*	Coefficient (95% CI)	*p*-value	*N*
*Type of explant -* Combined	−79.63 (−229.71,70.45)	0.30	55	−137.44 (−371.01,96.13)	0.26	41
Isolated	−85.52 (−240.25,69.21)	0.28	55	−20.87 (−258.50,216.76)	0.86	41
*Age*	0.55 (−3.49,4.59)	0.79	55	2.83 (−3.20,8.86)	0.36	41
*Chronic kidney disease*	2.68 (−109.02,114.37)	0.96	55	7.75 (−158.49,173.99)	0.93	41
*Diabetes mellitus*	−72.72 (−196.39,50.96)	0.25	55	40.97 (−137.15,219.10)	0.65	41

	LV capillary density mid layer			LV capillary density sub endocardium		
Variable	Coefficient (95% CI)	*p*-value	*N*	Coefficient (95% CI)	*p*-value	*N*
*Type of explant -* Combined	−104.19 (−315.30,106.92)	0.34	41	−131.25 (−328.41,65.92)	0.20	41
Isolated	−130.43 (−345.20, 84.34)	0.24	41	−115.70 (−316.28,84.89)	0.27	41
*Age*	2.44 (−2.96,7.85)	0.38	41	3.81 (−1.17,8.79)	0.14	41
*Chronic kidney disease*	−5.49 (−154.40,143.43)	0.94	41	80.69 (−56.68,218.06)	0.26	41
*Diabetes mellitus*	−12.49 (−172.41,147.43)	0.88	41	−151.78 (−294.07,−9.50)	0.043	41

Abbreviations: CI, confidence intervals; RV, Right Ventricle; LV, left ventricle.

β coefficients represents change in capillary density (microvessels/mm^2^) per unit increase in predictor variable. For continuous variables (age), coefficients represent change per 1-unit increase. For categorical variables (PH subtype, DM, CKD), coefficients represent the difference compared to the reference category (no PH, no diabetes, no CKD).

### Multivariate analysis of histopathologic predictors

To evaluate independent associations between PH subtype and capillary rarefaction, we performed multivariable linear regression models controlling for age, DM, and CKD as covariates without adjustment for center ( Table 4). In the RV, both PH subtypes were independently associated with lower microvessels/mm^2^ in mid-myocardial capillary density, specifically when compared to patients without PH (CpcPH: β = −228.05, 95% CI: −426.55 to −29.55; *p* = 0.031; IpcPH: β = −237.61, 95% CI: −459.88 to −15.35; *p* = 0.043) after controlling for covariates. No other statistically significant associations were observed between PH subtype and RV capillary density in other myocardial layers. Table S7 displays this multivariate analysis but further adjusted for center.

**Table 4:** Multivariable Linear Regression Models

	RV capillary density mean (*N* = 55)		RV capillary density sub epicardium (*N* = 42)	
Variable	Coefficient (95% CI)	*p*-value	Coefficient (95% CI)	*p*-value
*Type of explant -* Combined	−62.18 (−217.92,93.57)	0.44	−140.92 (−366.51,84.68)	0.23
Isolated	−63.20 (−231.28,104.89)	0.46	−149.49 (−402.09,103.12)	0.25
*Age*	2.13 (−2.26,6.53)	0.35	6.44 (−0.04,12.93)	0.06
*Chronic kidney disease*	86.08 (−36.35,208.52)	0.17	75.88 (−101.76,253.51)	0.41
*Diabetes mellitus*	31.93 (−94.01,157.87)	0.62	42.22 (−127.74,212.17)	0.63

	RV capillary density mid layer (*N* = 42)		RV capillary density sub endocardium (*N* = 43)	
Variable	Coefficient (95% CI)	*p*-value	Coefficient (95% CI)	*p*-value

*Type of explant -* Combined	−228.05 (−426.55,−29.55)	0.031	−153.76 (−402.00, 94.48)	0.23
Isolated	−237.61 (−459.88,−15.35)	0.043	−133.03 (−413.59,147.53)	0.36
*Age*	0.14 (−5.57,5.84)	0.96	−0.22 (−7.56,7.12)	0.95
*Chronic kidney disease*	132.59 (−23.72,288.89)	0.11	132.26 (−69.43,333.96)	0.21
*Diabetes mellitus*	58.57 (−90.98,208.11)	0.45	18.90 (−173.43,211.24)	0.85

	LV capillary density mean (*N* = 55)		LV capillary density sub epicardium (*N* = 41)	
Variable	Coefficient (95% CI)	*p*-value	Coefficient (95% CI)	*p*-value
*Type of explant -* Combined	−81.02 (−245.30,83.25)	0.34	−116.10 (−370.90,138.70)	0.38
Isolated	−85.91 (−263.20,91.38)	0.35	6.62 (−268.35,281.59)	0.96
*Age*	0.28 (−4.35,4.91)	0.91	2.46 (−4.40,9.33)	0.49
*Chronic kidney disease*	30.72 (−98.42,159.86)	0.64	−23.23 (−216.79,170.33)	0.82
*Diabetes mellitus*	−72.01 (−204.85,60.83)	0.29	28.22 (−163.11,219.56)	0.77

	LV capillary density mid layer (*N* = 41)		LV capillary density sub endocardium (*N* = 41)	
Variable	Coefficient (95% CI)	*p*-value	Coefficient (95% CI)	*p*-value
*Type of explant -* Combined	−95.24 (−325.95,135.48)	0.42	−133.17 (−319.65,53.32)	0.17
Isolated	−126.80 (−375.78,122.18)	0.33	−152.66 (−353.91,48.58)	0.15
*Age*	2.07 (−4.15,8.28)	0.52	4.11 (−0.91,9.13)	0.12
*Chronic kidney disease*	18.91 (−156.36,194.17)	0.83	125.84 (−15.83,267.50)	0.09
*Diabetes mellitus*	−31.52 (−204.77,141.73)	0.72	−207.16 (−347.20,−67.13)	0.006

Abbreviations: CI, confidence intervals; RV, right ventricle; LV, left ventricle.

β coefficients represents change in capillary density (microvessels/mm^2^) per unit increase in predictor variable. For continuous variables (age), coefficients represent change per 1-unit increase. For categorical variables (PH subtype, DM, CKD), coefficients represent difference compared to reference category (no PH, no diabetes, no CKD).

In the LV, DM remained significantly associated with 207.16 microvessels/mm^2^ lower sub-endocardial capillary density (β = −207.16, 95% CI: −347.20 to −67.13; *p* = 0.006). PH subtype was not associated with LV capillary density in any region after covariate adjustment.

#### Multivariable analysis of right ventricle fibrosis

To evaluate whether RV fibrosis differed between PH subtypes after accounting for clinical confounders, we performed multivariable linear regression adjusting for age, sex, DM, etiology of heart failure, RV end-diastolic pressure, and right atrial pressure (Table 5).

**Table 5:** Multivariate Linear Regression for RV Fibrosis and RV Collagen: Myocyte

	RV % fibrosis *N* = 51		RV collagen: myocyte ratio *N* = 51	
Variable	Coefficient (95% CI)	*p*-value	Coefficient (95% CI)	*p*-value
*Type of explant-* Combined	−0.90 (−1.77,−0.03)	0.049	−0.18 (−0.43,0.08)	0.19
Isolated	−0.85 (−1.82,0.12)	0.10	−0.13 (−0.41,0.16)	0.38
*Age*	−0.01 (−0.03,0.02)	0.55	−0.01 (−0.01,0.00)	0.07
*Gender -* Male	0.30 (−0.33,0.94)	0.36	0.12 (−0.07,0.31)	0.22
*Diabetes mellitus*	−0.23 (−0.88,0.41)	0.48	−0.10 (−0.28,0.09)	0.33
*Etiology of heart failure -* Congenital	0.36 (−0.79,1.50)	0.55	0.09 (−0.25,0.42)	0.61
Ischemic	0.93 (−0.31,2.17)	0.15	0.15 (−0.21,0.51)	0.42
Myocarditis	−0.09 (−1.78,1.60)	0.92	−0.15 (−0.65,0.35)	0.56
Other	0.69 (−0.29,1.66)	0.18	0.14 (−0.15,0.42)	0.35
Valvular	−0.31 (−2.48,1.87)	0.78	−0.09 (−0.73,0.55)	0.78
*RV diastolic pressure*	0.00 (−0.07,0.07)	0.95	0.01 (−0.01,0.03)	0.42
*RA pressure*	0.01 (−0.06,0.07)	0.83	−0.01 (−0.03,0.01)	0.54

Abbreviation: CI, confidence intervals; RV, right ventricle.

Multivariable linear regression analysis for RV fibrosis. β coefficients represent the change in 10 times the original unit of RV fibrosis (%) per unit change in each continuous variable; for categorical variables, are differences between the specific category versus reference categories (i.e., no PH, female sex, no diabetes, University of Florida, and arrhythmia etiology). For example, the combined PH group has RV fibrosis 0.09% (come from 0.90 divided by 10) lower than the no PH group after adjusting for covariates.

In the primary pooled analysis, combined PH demonstrated lower RV fibrosis compared to no PH (β = −0.90, 95% CI:−1.77 to −0.03, *p* = 0.049), after covariate adjustment. Since we inflated RV fibrosis by ten times in the model to accommodate the small magnitude of percentage values for model convergence purpose, the coefficient was interpreted as the combined PH group has RV fibrosis 0.09% (come from 0.90 divided by 10) lower than the no PH group. In sensitivity analysis adjusted for institution (Table S8), this group difference was no longer significant (β = −0,74, 95% CI:−1.77 to 0.28, *p* = 0.16). RV collagen: myocyte ratio similarly showed no group effects after adjusting for both covariates and institution. Variance inflation factors were <5 for all variables, confirming no multicollinearity.

## Discussion

In this multicenter analysis of explanted human hearts, we found that RV capillary rarefaction was associated with impaired RV function; however, RV fibrosis was not. This finding suggests that CMD may represent a distinct tissue-level correlate of RV failure across PH subtypes in LV failure. Preserved microvascular architecture was linked to more favorable RV functional parameters, including higher TAPSE and TAPSE/sPAP ratio. In contrast, RV interstitial fibrosis did not correlate with these measures, suggesting that fibrotic remodeling may reflect cumulative injury mechanisms not directly aligned with hemodynamic load.

### Microvascular rarefaction and localized to the right ventricle mid-myocardial layer

Multivariable analysis revealed that both IpcPH and CpcPH were independently associated with reduced RV mid-myocardial capillary density, even after accounting for age, DM and CKD. This selective vulnerability suggests a regional predilection for microvascular dropout within the RV. Possible contributors may include perfusion gradients across myocardial layers, differential oxygen demand, and shear stress-related endothelial injury. Additional studies are needed to test these hypotheses.

### Fibrosis and pulmonary hypertension: a complex heterogenous relationship

We initially hypothesized that both interstitial fibrosis and capillary rarefaction would be associated with impaired RV function. While fibrosis is a well-established feature of maladaptive ventricular remodeling, we did not find this association in our analysis. After multivariable adjustment, we found no significant difference in RV fibrosis between PH groups. Even after accounting for ischemic versus non-ischemic etiology, alternative drivers of fibrosis- such as primary cardiomyopathies, arrythmia-related remodeling, and degree of coronary disease burden-likely contributed to the heterogeneity in fibrotic patterns. The counterintuitive finding of higher RV fibrosis in patients without PH (*p* = 0.024, Table 2) further underscores the complex, multifactorial nature of myocardial fibrosis in end-stage heart failure, highlighting the challenge of detecting disease-specific patterns in heterogenous populations.

### Integration with prior literature

Our findings align with prior HFpEF studies in which myocardial capillary rarefaction, rather than fibrosis, has been linked to exercise intolerance and adverse remodeling.^21^ Similarly, in CAV, microvascular rarefaction has been associated with diastolic dysfunction and graft failure, underscoring the importance of microvascular integrity.^16^ Preclinical PH models demonstrate that sustained pressure overload and inflammatory signaling can drive endothelial dropout, impaired angiogenesis, and regional perfusion mismatch.^10^, ^22^, ^23^ While these models support a causal link between hemodynamic load and rarefaction, our cohort did not show a clear association between current RV load and capillary density, suggesting that in advanced human disease, rarefaction may persist or progress independent of hemodynamic burden. The limited correlation between RV and LV capillary densities in our data further suggests that RV microvascular injury in PH may evolve through distinct pathophysiologic pathways—an area that warrant deeper investigation.

Our 2-dimensional CD31-based digital pathology quantifies capillary density but cannot capture three-dimensional (3D) network architecture. Ichimura et al (2024) applied 3D imaging in pressure-overloaded mice and human PAH heart tissue,^23^ revealing a paradox that challenges simple interpretations of our findings. They found complex remodeling—capillaries became tortuous, shorter, thicker, and highly branched—yet overall capillary density remained preserved in Group 1 PAH patients with severe pressure overload. In contrast, our Group 2 PH cohort demonstrated new rarefaction despite experiencing comparatively lower RV afterload. This apparent contradiction—where higher pressure preserves density while lower pressure shows loss-suggests pressure overload alone cannot explain capillary remodeling patterns and raises several mechanistic possibilities.

First the systemic metabolic milieu in Group 2 PH may fundamentally differ from Group 1. Group 2 PH patients carry higher burden of diabetes, hypertension, and obesity^13^, ^24^-comorbidities that independently drive endothelial dysfunction and microvascular rarefaction across multiple organs. These systemic factors may override or prevent the compensatory angiogenic responses to pressure overload that characterized Group 1 PAH. Second, methodological bias cannot be excluded: 2D histological sampling may systematically underestimate capillary density in highly tortuous remodeling networks typical of Group 1 PAH, while more accurately capturing true loss in Group 2 PH Third, Group 2 PH may represent a distinct “metabolic-microvascular” phenotype where LV dysfunction, volume overload, and systemic inflammation create an environment hostile to vascular maintenance, independent of mechanical stress. Whether the net rarefaction we observed in Group 2 PH reflects true capillary loss, 3D remodeling patterns detectable only with 2D sampling, or a biologically distinct disease mechanism requires future studies combining both methodologies in the same cohort. Until this paradox is resolved through direct comparative studies, our finding should be interpreted as identifying an association between mid-myocardial capillary density and RV dysfunction in Group 2 PH, rather than providing a causal pathway applicable across all PH subtypes.

### Mechanistic interpretation

Our exploratory findings suggest that microvascular rarefaction in PH likely reflects a convergence of endothelial loss, angiogenic failure, and regional perfusion mismatch, compounded by metabolic and inflammatory stressors unique to the RV in chronic pressure overload.^10^, ^22^ The selective vulnerability of the mid-myocardial layer may relate to regional perfusion patterns, oxygen demand gradients, or microvascular shear stress alterations. The absence of strong RV-LV correlations in capillary density suggest that PH-associated RV microvascular injury may not simply reflect LV pathology but rather implies unique and independent ventricular environments and remodeling.^25^

This ventricular independence has important implications for both phenotyping and treatment. It raises the possibility that RV- specific microvascular vulnerability is worthy of consideration independent of addressing LV pathology, especially in PH subtypes where the RV is uniquely affected. This concept would support the development of targeted interventions—whether pharmacologic, mechanical, or regenerative—that directly addresses RV coronary microcirculation integrity as part of advanced heart failure and PH management.

**Figure 5:**
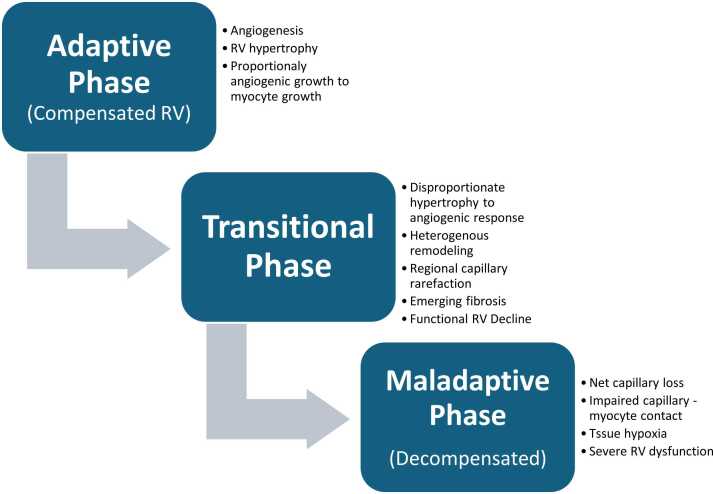
*Proposed mechanism of RV microvascular remodeling progression.* Legend: Proposed conceptual framework of adaptive, transitional, and maladaptive phases of RV microvascular remodeling. This schematic is hypothesis-generating; our cross-sectional data captures only end-stage disease (Phase 3) and cannot validate earlier stages. RV, right ventricle.

Our cross-sectional design in end-stage explanted hearts precludes determination of whether microvascular loss is cause or consequence of RV failure, or both. We propose a conceptual framework (Figure 5) with three phases: (1) adaptive phase (compensated RV)-preserved or increased capillary density maintains cardiomyocyte-capillary contact despite increased afterload; (2) transition phase: heterogenous microvascular response with regional rarefaction, imbalanced angiogenesis relative to cardiomyocyte hypertrophy, declining RV function, and emerging histological changes (regional density loss, early fibrosis); (3) maladaptive phase (decompensated RV): net microvascular rarefaction, loss of capillary-myocyte contact, tissue hypoxia, and severe RV impairment with marked capillary density loss and fibrosis. In this framework, microvascular rarefaction may both result from metabolic stress and angiogenic failure while simultaneously contributing to ischemia and contractile dysfunction.

Our finding that diabetes was independently associated with LV subendocardial capillary loss (β = −207, *p* 0.006) is mechanistically relevant because diabetic microvascular dysfunction is a systemic process and affects the entire heart.^26^ Prior work has shown that diabetic myocardium exhibits pronounced capillary rarefaction and pericyte loss compared to non-diabetic hearts.^27^ Thus, diabetes likely reduces the angiogenic reserve in both ventricles. In Group 2 PH, elevated left atrial pressure- whether from systolic/diastolic dysfunction or valvular disease- promotes pulmonary vascular remodeling and increased RV afterload. However, we did not detect a significant association between diabetes and RV capillary density in our cohort (Table 4), despite the clear LV effect. Whether this reflects true RV-specific resilience, insufficient statistical power (*n* = 23 diabetics), or masking by competing pathologies in end-stage heart failure requires larger studies. Mohammed et al (2015) found that HFpEF patients, in whom diabetes is common, had significantly lower LV density than controls.^13^ By analogy, diabetes-associated rarefaction in our explanted hearts may signify a shared substrate of microvascular disease that worsens biventricular function. The “double hit” hypothesis of diabetes impairing both ventricles remains speculative based on our data and would require validation before suggesting that therapies addressing diabetic endothelial dysfunction could preserve RV microvascular adaptation in PH.

### Clinical implications

Our results highlight CMD as a possible histopathologic marker of RV maladaptation that is not readily detectable by standard echocardiography or invasive hemodynamic assessment yet shows strong correlation with functional RV measures. This suggests a potential role for emerging imaging biomarkers—such as cardiac MRI perfusion mapping, positron emission tomography, or myocardial contrast echocardiography—in detecting early RV microvascular injury.^28^, ^29^, ^30^ These modalities could inform transplant risk stratification, identify RV-predominant PH sub-phenotypes, and guide the development of RV-specific microvascular protective or regenerative therapies.

The stage-dependence of microvascular remodeling suggests therapies targeting angiogenesis may benefit early adaptive phases but could be futile once maladaptive rarefaction is established. Biomarkers of early RV microvascular dysfunction—including circulating angiogenic factors and advanced tissue characterization via perfusion cardiac MRI- are needed to identify optimal intervention windows.

## Limitations

This study had several limitations. First, the sample size was modest, and the observational design limits causal inference. Second, echocardiographic and hemodynamic data were not concurrent with explant tissue collection, introducing potential measurement error. Third, while exact timing of functional assessments relative to tissue collection was unavailable, the median listing-to transplant interval of 1.6 months, combined with typical pre-listing evaluation timing, suggest most correlations reflect concurrent pathophysiology. Fourth, all tissue samples were derived from end-stage heart failure patients, which cannot account for earlier disease-specific patterns. Fifth, no control group of histologically normal ventricular tissue was available for comparison. Sixth, a single timepoint in advanced disease and cannot establish the temporal sequence of microvascular changes. Longitudinal studies with serial imaging (e.g., cardiac MRI, PET imaging) and serum biomarkers are needed to define the trajectory from adaptive to maladaptive remodeling. Finally, the heterogeneity of the cohort-particularly in Group 2 PH—introduces competing pathologies that may confound disease -specific associations.

### Future directions

Our findings underscore coronary microvascular rarefaction as a measurable and potentially modifiable contributor to RV failure in PH. Defining CMD phenotypes at the tissue level lays the groundwork for validating noninvasive imaging surrogates, refining PH risk stratification, and developing targeted therapies aimed at preserving RV microcirculation and function. Future studies should leverage prospective, multi-modality approaches to better delineate temporal relationships between microvascular injury, fibrosis, and RV failure.

## Conclusion

This multicenter proof-of-concept study establishes capillary rarefaction as a measurable and potentially modifiable histopathologic phenotype of RV failure in PH. Our study provides a foundation for future mechanistic studies investigating CMD as a therapeutic target in right ventricular failure and supports the development of noninvasive imaging biomarkers for early detection of RV microvascular injury.

## Conflicts of Interest statement

The authors declare the following financial interests/personal relationships, which may be considered as potential competing interests: Zhining Ou reports financial support and statistical analysis were provided by the National Institutes of Health. Katharine Clapham reports a relationship with Amgen Inc., that includes: consulting or advisory. Katherine Clapham reports a relationship with Eir ventures that includes: consulting or advisory. Katharine Clapham reports a relationship with Tectonic Therapeutics, Inc., that includes: consulting or advisory. Katharine Clapham reports a relationship with United Therapeutics Corporation that includes: board membership. John Ryan reports a relationship with United therapeutics that includes: Speaker and honoraria. John Ryan reports a relationship with United therapeutics that includes: Speaker and honoraria. John Ryan reports a relationship with Liquida that includes: Speaker and honoraria. John Ryan reports a relationship with Merk that includes: Speaker and honoraria. John Ryan reports a relationship with Johnson and Johnson that includes: Speaker and honoraria. John Ryan reports a relationship with Gossamer Bio that includes: Speaker and honoraria. John Ryan reports a relationship with Kiniksa that includes: Speaker and honoraria. If there are other authors, they declare that they have no known competing financial interests or personal relationships that could have appeared to influence the work reported in this paper.

## Financial support

This investigation was supported by the University of Utah Study Design and Biostatistics Center, with funding in part from the National Center for Research Resources and the National Center for Advancing Translational Sciences, National Institutes of Health, through Grant UL1TR002538 (formerly 5UL1TR001067-05, 8UL1TR000105, and UL1RR025764).

## Footnotes

Appendix A Supplementary data associated with this article can be found in the online version at doi:10.1016/j.jhlto.2025.100459.
