# Supplementary material for: The right kind of rarefaction: Coronary microvascular remodeling in right ventricle failure
Source: JHLT Open. 2025 Dec 2;11:100459. doi: 10.1016/j.jhlto.2025.100459 (PMC12816859; doi:10.1016/j.jhlto.2025.100459)
Supplement: Supplementary file 1 — Supplementary material [file mmc1.docx]

**Supplemental Data**

**Table S1. Summary of demographics and clinical characteristics stratification by centers.**

| **Variable** | **Total (N=57)** | **University of Florida (N=16)** | **University of Pennsylvania (N=10)** | **University of Utah (N=31)** | **P-value** |
| --- | --- | --- | --- | --- | --- |
| **Gender -** Female | 17(30%) | 4(25%) | 3(30%) | 10(32%) | 0.92^f^ |
| Male | 40(70%) | 12(75%) | 7(70%) | 21(68%) | - |
| **Age -** Mean(SD) | 52 (14) | 50 (13) | 47 (17) | 54 (13) | 0.28^k^ |
| Median (IQR) | 56 (45, 62) | 54 (42, 59) | 52 (32, 58) | 58 (46, 64) | - |
| Range | (21, 70) | (24, 65) | (22, 69) | (21, 70) | - |
| **Race -** American Indian | 1(1.8%) | 0(0%) | 0(0%) | 1(3.2%) | 0.008^f^ |
| Asian | 1(1.8%) | 1(6.2%) | 0(0%) | 0(0%) | - |
| Black | 7(12%) | 2(12%) | 4(40%) | 1(3.2%) | - |
| Hispanic/Latino | 10(18%) | 5(31%) | 2(20%) | 3(9.7%) | - |
| White | 38(67%) | 8(50%) | 4(40%) | 26(84%) | - |
| **BMI -** 18.5-24.9 | 17(30%) | 2(12%) | 5(50%) | 10(32%) | 0.24^f^ |
| 25-29.9 | 24(42%) | 11(69%) | 3(30%) | 10(32%) | - |
| 30-34.9 | 10(18%) | 2(12%) | 1(10%) | 7(23%) | - |
| 35 or higher | 6(11%) | 1(6.2%) | 1(10%) | 4(13%) | - |
| **Etiology of heart failure -** Arrhythmia | 5(8.8%) | 0(0%) | 0(0%) | 5(16%) | 0.046^f^ |
| Congenital | 9(16%) | 3(19%) | 0(0%) | 6(19%) | - |
| Ischemic | 11(19%) | 7(44%) | 1(10%) | 3(9.7%) | - |
| Myocarditis | 3(5.3%) | 1(6.2%) | 1(10%) | 1(3.2%) | - |
| Other | 28(49%) | 5(31%) | 8(80%) | 15(48%) | - |
| Valvular | 1(1.8%) | 0(0%) | 0(0%) | 1(3.2%) | - |
| **pacemaker implanted -** No | 34(60%) | 10(62%) | 4(40%) | 20(65%) | 0.44^f^ |
| Yes | 23(40%) | 6(38%) | 6(60%) | 11(35%) | - |
| **icd implanted -** No | 14(25%) | 2(12%) | 4(40%) | 8(26%) | 0.26^f^ |
| Yes | 43(75%) | 14(88%) | 6(60%) | 23(74%) | - |
| **Hypertension -** No | 28(49%) | 4(25%) | 7(70%) | 17(55%) | 0.050^f^ |
| Yes | 29(51%) | 12(75%) | 3(30%) | 14(45%) | - |
| **Diabetes mellitus -** No | 42(74%) | 9(56%) | 9(90%) | 24(77%) | 0.16^f^ |
| Yes | 15(26%) | 7(44%) | 1(10%) | 7(23%) | - |
| **Latest HbA1c -** Mean(SD) | 5.8 (0.96) | 5.7 (1.2) | 5.6 (0.48) | 6.0 (0.93) | 0.52^k^ |
| Median (IQR) | 5.7 (5.3, 6.3) | 5.5 (5.1, 6.8) | 5.6 (5.2, 5.8) | 5.7 (5.4, 6.2) | - |
| Range | (3.5, 8.6) | (3.5, 7.4) | (4.8, 6.3) | (4.9, 8.6) | - |
| **Chronic kidney disease -** No | 30(53%) | 11(69%) | 6(60%) | 13(42%) | 0.20^f^ |
| Yes | 27(47%) | 5(31%) | 4(40%) | 18(58%) | - |
| **Hyperlipidemia -** No | 28(49%) | 6(38%) | 5(50%) | 17(55%) | 0.59^f^ |
| Yes | 29(51%) | 10(62%) | 5(50%) | 14(45%) | - |
| **Peripheral vascular disease -** No | 51(89%) | 15(94%) | 8(80%) | 28(90%) | 0.61^f^ |
| Yes | 6(11%) | 1(6.2%) | 2(20%) | 3(9.7%) | - |
| **OSA/OHVS -** No | 43(75%) | 15(94%) | 8(80%) | 20(65%) | 0.07^f^ |
| Yes | 14(25%) | 1(6.2%) | 2(20%) | 11(35%) | - |
| **use of inotrope prior to transplant -** No | 12(21%) | 4(25%) | 2(20%) | 6(19%) | 0.91^f^ |
| Yes | 45(79%) | 12(75%) | 8(80%) | 25(81%) | - |
| **use of vasopressor prior to transplant -** No | 38(67%) | 12(75%) | 8(80%) | 18(58%) | 0.38^f^ |
| Yes | 19(33%) | 4(25%) | 2(20%) | 13(42%) | - |
| **Use of MCS -** Checked | 21(37%) | 2(12%) | 6(60%) | 13(42%) | 0.028^f^ |
| Unchecked | 36(63%) | 14(88%) | 4(40%) | 18(58%) | - |
| **time on waiting list -** > 1 year | 6(11%) | 3(19%) | 0(0%) | 3(9.7%) | 0.45^f^ |
| 1-3 months | 13(23%) | 5(31%) | 1(10%) | 7(23%) | - |
| 1 week to 1 month | 15(26%) | 3(19%) | 2(20%) | 10(32%) | - |
| 3-6 months | 11(19%) | 3(19%) | 3(30%) | 5(16%) | - |
| 6-12 months | 7(12%) | 2(12%) | 1(10%) | 4(13%) | - |
| less than 1 week | 5(8.8%) | 0(0%) | 3(30%) | 2(6.5%) | - |
| **Waitlist decompensation -** more than 3 (transplant from outpatient) | 1(1.8%) | 0(0%) | 0(0%) | 1(3.2%) | 0.055^f^ |
| none (came in from outpatient) | 13(23%) | 2(12%) | 5(50%) | 6(19%) | - |
| one (came in from outpatient) | 7(12%) | 0(0%) | 0(0%) | 7(23%) | - |
| one (lead to transplant) | 29(51%) | 12(75%) | 4(40%) | 13(42%) | - |
| three (final led to transplant) | 1(1.8%) | 0(0%) | 0(0%) | 1(3.2%) | - |
| two (final lead to transplant) | 5(8.8%) | 2(12%) | 0(0%) | 3(9.7%) | - |
| two (transplant from outpatient) | 1(1.8%) | 0(0%) | 1(10%) | 0(0%) | - |

Missing values: Latest HbA1c=0 (0%)/0 (0%)/1 (3.2%).

BMI, body mass index; ICD, implantable cardioverter-defibrillator; MCS, mechanical circulatory support; OHVS, obesity hypoventilation syndrome; OSA, obstructive sleep apnea

**Figure S1. Boxplots of LV/RV capillary density, percent fibrosis and Collagen:Myocyte ratio stratified by PH subtypes.**


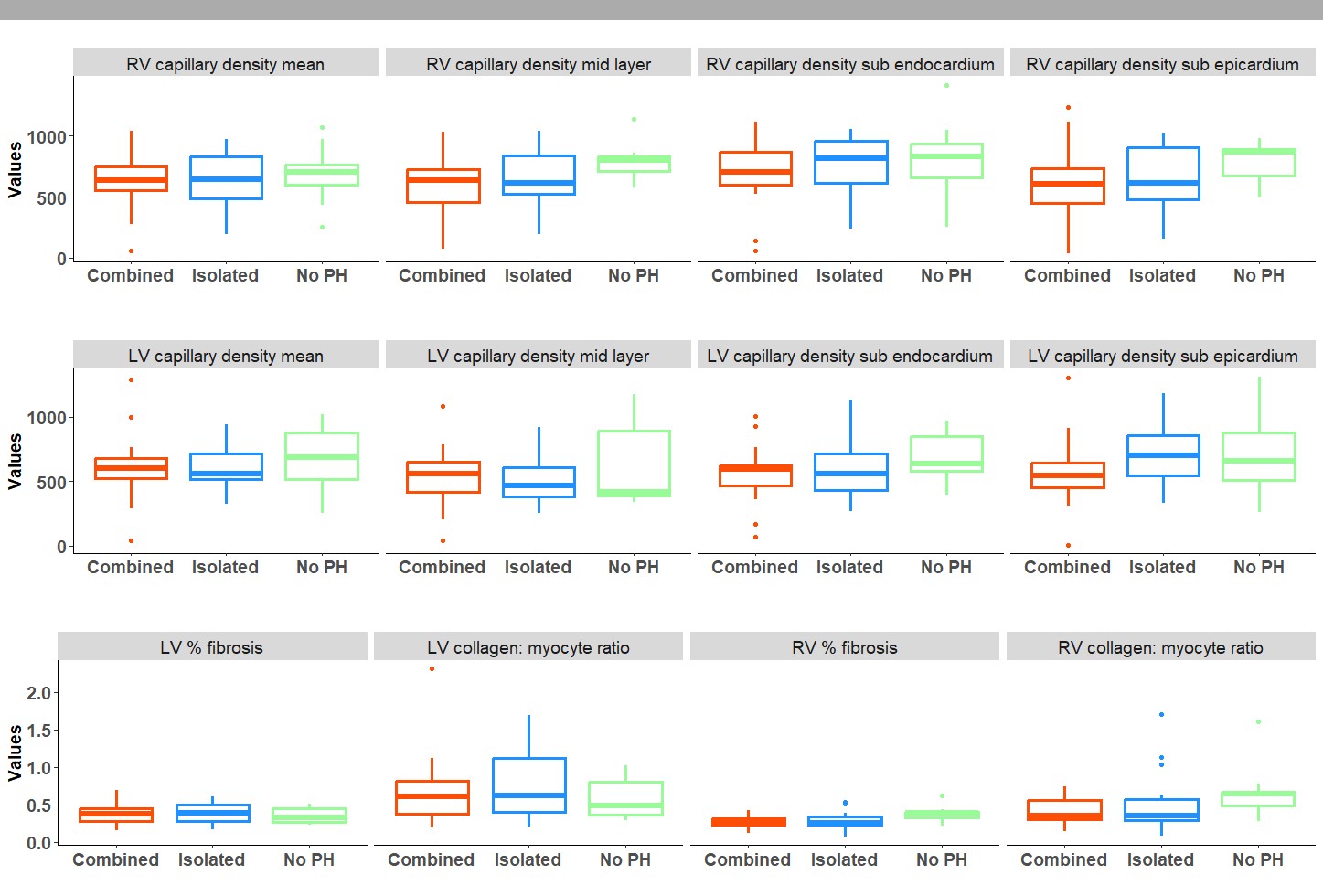


**Table S2. Histopathology characteristics stratification by centers**

| **Variable** | **Total (N=57)** | **University of Florida (N=16)** | **University of Pennsylvania (N=10)** | **University of Utah (N=31)** | **P-value** |
| --- | --- | --- | --- | --- | --- |
| **RV capillary density mean -** Mean(SD) | 653 (204) | 644 (170) | 477 (274) | 717 (159) | 0.025^k^ |
| Median (IQR) | 653 (549, 761) | 642 (520, 756) | 526 (260, 639) | 701 (590, 846) | - |
| Range | (57, 1067) | (432, 1038) | (57, 923) | (429, 1067) | - |
| **RV capillary density sub epicardium -** Mean(SD) | 663 (258) | 632 (207) | 406 (241) | 798 (203) | <0.001^k^ |
| Median (IQR) | 636 (471, 881) | 578 (464, 736) | 428 (300, 500) | 791 (638, 979) | - |
| Range | (40, 1236) | (407, 1111) | (40, 890) | (419, 1236) | - |
| **RV capillary density mid layer -** Mean(SD) | 656 (225) | 677 (227) | 493 (255) | 715 (180) | 0.09^k^ |
| Median (IQR) | 658 (522, 802) | 647 (544, 797) | 522 (391, 621) | 701 (613, 823) | - |
| Range | (71, 1135) | (337, 1036) | (71, 833) | (427, 1135) | - |
| **RV capillary density sub endocardium -** Mean(SD) | 745 (272) | 788 (201) | 571 (395) | 805 (209) | 0.30^k^ |
| Median (IQR) | 765 (601, 913) | 886 (617, 966) | 569 (240, 948) | 810 (694, 896) | - |
| Range | (59, 1417) | (517, 1049) | (59, 1113) | (441, 1417) | - |
| **RV % fibrosis -** Mean(SD) | 0.30 (0.10) | 0.32 (0.06) | 0.29 (0.16) | 0.29 (0.10) | 0.17^k^ |
| Median (IQR) | 0.27 (0.24, 0.38) | 0.33 (0.27, 0.38) | 0.25 (0.20, 0.28) | 0.26 (0.22, 0.38) | - |
| Range | (0.07, 0.62) | (0.24, 0.42) | (0.12, 0.62) | (0.07, 0.51) | - |
| **RV collagen: myocyte ratio -** Mean(SD) | 0.49 (0.31) | 0.49 (0.14) | 0.53 (0.50) | 0.48 (0.31) | 0.35^k^ |
| Median (IQR) | 0.37 (0.31, 0.63) | 0.49 (0.36, 0.61) | 0.35 (0.24, 0.41) | 0.35 (0.29, 0.64) | - |
| Range | (0.08, 1.7) | (0.31, 0.74) | (0.14, 1.6) | (0.08, 1.7) | - |
| **LV capillary density mean -** Mean(SD) | 618 (209) | 508 (138) | 576 (330) | 687 (163) | 0.006^k^ |
| Median (IQR) | 608 (516, 708) | 535 (422, 594) | 546 (445, 669) | 657 (558, 762) | - |
| Range | (42, 1289) | (258, 708) | (42, 1289) | (392, 1020) | - |
| **LV capillary density sub epicardium -** Mean(SD) | 647 (268) | 619 (253) | 493 (336) | 740 (213) | 0.024^k^ |
| Median (IQR) | 630 (470, 793) | 560 (465, 657) | 427 (315, 576) | 694 (612, 878) | - |
| Range | (9.3, 1313) | (332, 1305) | (9.3, 1182) | (448, 1313) | - |
| **LV capillary density mid layer -** Mean(SD) | 545 (240) | 489 (186) | 348 (170) | 676 (229) | 0.002^k^ |
| Median (IQR) | 471 (391, 668) | 417 (391, 519) | 360 (262, 458) | 655 (537, 791) | - |
| Range | (44, 1175) | (253, 908) | (44, 629) | (338, 1175) | - |
| **LV capillary density sub endocardium -** Mean(SD) | 592 (225) | 466 (207) | 633 (267) | 659 (188) | 0.023^k^ |
| Median (IQR) | 586 (468, 736) | 459 (327, 567) | 760 (564, 768) | 601 (556, 719) | - |
| Range | (74, 1133) | (171, 971) | (74, 930) | (384, 1133) | - |
| **LV % fibrosis -** Mean(SD) | 0.37 (0.13) | 0.43 (0.10) | 0.31 (0.09) | 0.36 (0.14) | 0.029^k^ |
| Median (IQR) | 0.37 (0.27, 0.46) | 0.45 (0.39, 0.48) | 0.28 (0.26, 0.36) | 0.32 (0.24, 0.44) | - |
| Range | (0.16, 0.70) | (0.24, 0.58) | (0.16, 0.47) | (0.17, 0.70) | - |
| **LV collagen: myocyte ratio -** Mean(SD) | 0.68 (0.42) | 0.77 (0.31) | 0.48 (0.21) | 0.69 (0.50) | 0.09^k^ |
| Median (IQR) | 0.58 (0.38, 0.85) | 0.67 (0.57, 0.87) | 0.40 (0.35, 0.58) | 0.48 (0.31, 0.89) | - |
| Range | (0.19, 2.3) | (0.33, 1.4) | (0.19, 0.89) | (0.21, 2.3) | - |

Missing values: RV capillary density mean =1 (6.2%)/0 (0%)/1 (3.2%), RV capillary density sub epicardium =3 (19%)/1 (10%)/11 (35%), RV capillary density mid layer =3 (19%)/1 (10%)/11 (35%), RV capillary density sub endocardium =3 (19%)/0 (0%)/11 (35%), RV % fibrosis =1 (6.2%)/0 (0%)/1 (3.2%), RV collagen: myocyte ratio =1 (6.2%)/1 (10%)/0 (0%), LV capillary density mean =1 (6.2%)/0 (0%)/1 (3.2%), LV capillary density sub epicardium =3 (19%)/1 (10%)/12 (39%), LV capillary density mid layer =3 (19%)/1 (10%)/12 (39%), LV capillary density sub endocardium =3 (19%)/1 (10%)/12 (39%), LV % fibrosis =1 (6.2%)/0 (0%)/1 (3.2%), LV collagen: myocyte ratio =1 (6.2%)/1 (10%)/0 (0%).

^k^ Kruskal-Wallis test.

**Table S3. Pairwise partial Spearman’s rank correlation coefficients, 95% CI, and p-values between the LV/RV cap density mean and the echo/cath. Controlled for by center.**

|  | **RV capillary density mean** |  | **LV capillary density mean** |  |
| --- | --- | --- | --- | --- |
|  | ρ_s_ **(95% CI)** | **p-value** | ρ_s_ **(95% CI)** | **p-value** |
| Left ventricular Ejection fraction LVEF | 0.05 (-0.25, 0.31) | 0.73 | 0 (-0.31, 0.32) | >0.99 |
| Right Atrial Pressure | -0.05 (-0.37, 0.29) | 0.77 | 0.24 (-0.06, 0.53) | 0.12 |
| Tricuspid regurgitation | 0.06 (-0.24, 0.37) | 0.71 | -0.13 (-0.47, 0.25) | 0.40 |
| RVSP | 0.02 (-0.3, 0.32) | 0.91 | 0.02 (-0.31, 0.33) | 0.88 |
| Tapse measurement | 0.47 (0.16, 0.68) | 0.002 | 0.04 (-0.27, 0.42) | 0.82 |
| LA dilation size | 0.26 (-0.03, 0.51) | 0.07 | -0.12 (-0.39, 0.2) | 0.41 |
| RA pressure | -0.03 (-0.3, 0.26) | 0.85 | -0.1 (-0.39, 0.17) | 0.47 |
| mean PAP | 0.02 (-0.24, 0.27) | 0.90 | -0.21 (-0.46, 0.05) | 0.13 |
| PCWP | 0.04 (-0.25, 0.31) | 0.75 | -0.14 (-0.38, 0.13) | 0.31 |
| Pulmonary Vascular Resistance | -0.12 (-0.46, 0.17) | 0.38 | -0.19 (-0.43, 0.11) | 0.18 |
| Cardiac output CO Fick | 0.1 (-0.2, 0.35) | 0.48 | -0.26 (-0.5, 0) | 0.06 |
| Cardiac Index CI Fick | 0.02 (-0.25, 0.29) | 0.87 | -0.36 (-0.6, -0.09) | 0.009 |
| Mixed Venous O2 saturation | -0.07 (-0.41, 0.26) | 0.62 | -0.14 (-0.41, 0.12) | 0.33 |
| Tapse/sPAP | 0.28 (-0.05, 0.54) | 0.09 | 0.03 (-0.27, 0.38) | 0.84 |

|  | **RV % fibrosis** |  | **LV % fibrosis** |  |
| --- | --- | --- | --- | --- |
|  | ρ_s_ **(95% CI)** | **p-value** | ρ_s_ **(95% CI)** | **p-value** |
| Left ventricular Ejection fraction LVEF | -0.12 (-0.42, 0.15) | 0.38 | 0.08 (-0.22, 0.35) | 0.59 |
| Right Atrial Pressure | -0.03 (-0.37, 0.3) | 0.87 | -0.13 (-0.43, 0.2) | 0.43 |
| Triscupid regurgitation | -0.04 (-0.34, 0.33) | 0.80 | -0.07 (-0.36, 0.25) | 0.65 |
| RVSP | 0.01 (-0.3, 0.35) | 0.96 | -0.07 (-0.33, 0.27) | 0.67 |
| Tapse measurement | 0 (-0.32, 0.33) | >0.99 | 0.12 (-0.23, 0.41) | 0.44 |
| LA dilation size | 0 (-0.32, 0.39) | 0.98 | -0.18 (-0.44, 0.19) | 0.23 |
| RA pressure | -0.07 (-0.36, 0.22) | 0.64 | 0.05 (-0.21, 0.3) | 0.71 |
| mean PAP | -0.24 (-0.47, 0.05) | 0.09 | 0.17 (-0.11, 0.41) | 0.23 |
| PCWP | -0.19 (-0.42, 0.08) | 0.18 | 0.14 (-0.13, 0.39) | 0.32 |
| Pulmonary Vascular Resistance | 0.03 (-0.26, 0.34) | 0.81 | -0.02 (-0.32, 0.29) | 0.86 |
| Cardiac output CO Fick | -0.16 (-0.46, 0.15) | 0.26 | 0.36 (0.1, 0.58) | 0.009 |
| Cardiac Index CI Fick | -0.05 (-0.35, 0.23) | 0.73 | 0.22 (-0.06, 0.47) | 0.12 |
| Mixed Venous O2 saturation | -0.02 (-0.26, 0.25) | 0.90 | 0.06 (-0.24, 0.34) | 0.68 |
| Tapse/sPAP | 0 (-0.32, 0.35) | >0.99 | 0.1 (-0.28, 0.41) | 0.56 |

CI, cardiac index; CO, cardiac output; LA, left atrium; LVEF, left ventricular ejection fraction; PAP, pulmonary artery pressure; PCWP, pulmonary capillary wedge pressure; RA, right atrium; RVSP, right ventricular systolic pressure; sPAP, systolic pulmonary artery pressure; TAPSE, tricuspid annular plane systolic excursion

**Table S4. Pairwise Spearman’s rank correlation coefficients, 95% CI, and p-values on cap density variables within LV/RV (unadjusted by center)**

|  | **RV capillary density mean** | **RV capillary density sub epicardium** | **RV capillary density mid layer** | **RV capillary density sub endocardium** | **RV % fibrosis** | **RV collagen: myocyte ratio** |
| --- | --- | --- | --- | --- | --- | --- |
| RV capillary density mean | - | 0.86 (0.69, 0.93) | 0.82 (0.63, 0.91) | 0.77 (0.59, 0.89) | 0.08 (-0.22, 0.36) | 0.04 (-0.25, 0.32) |
| RV capillary density sub epicardium | <0.001 | - | 0.62 (0.35, 0.81) | 0.46 (0.17, 0.72) | 0.24 (-0.09, 0.53) | 0.21 (-0.14, 0.50) |
| RV capillary density mid layer | <0.001 | <0.001 | - | 0.52 (0.19, 0.77) | 0.06 (-0.29, 0.37) | 0.04 (-0.34,0.36) |
| RV capillary density sub endocardium | <0.001 | 0.002 | <0.001 | - | 0.03 (-0.37, 0.39) | -0.01 (-0.38, 0.35) |
| RV % fibrosis | 0.59 | 0.13 | 0.72 | 0.85 | - | 0.98 (0.93, 1.00) |
| RV collagen: myocyte ratio | 0.76 | 0.20 | 0.79 | 0.95 | <0.001 | - |

|  | **LV capillary density mean** | **LV capillary density sub epicardium** | **LV capillary density mid layer** | **LV capillary density sub endocardium** | **LV % fibrosis** | **LV collagen: myocyte ratio** |
| --- | --- | --- | --- | --- | --- | --- |
| LV capillary density mean | - | 0.86 (0.72, 0.93) | 0.9 (0.79, 0.95) | 0.56 (0.27, 0.77) | -0.24 (-0.46, 0.03) | -0.2 (-0.45, 0.06) |
| LV capillary density sub epicardium | <0.001 | - | 0.77 (0.59, 0.88) | 0.22 (-0.12, 0.56) | -0.08 (-0.39, 0.25) | -0.03 (-0.32, 0.29) |
| LV capillary density mid layer | <0.001 | <0.001 | - | 0.37 (0.05, 0.64) | -0.24 (-0.52, 0.07) | -0.17 (-0.47, 0.17) |
| LV capillary density sub endocardium | <0.001 | 0.17 | 0.019 | - | -0.46 (-0.66, -0.19) | -0.47 (-0.67, -0.19) |
| LV % fibrosis | 0.08 | 0.64 | 0.14 | 0.003 | - | 0.98 (0.94, 1.00) |
| LV collagen: myocyte ratio | 0.14 | 0.85 | 0.29 | 0.002 | <0.001 | - |

**Table S5. Pairwise partial Spearman’s rank correlation coefficients, 95% CI, and p-values on cap density variables within LV/RV adjusted for by center.**

|  | **RV capillary density mean** | **RV capillary density sub epicardium** | **RV capillary density mid layer** | **RV capillary density sub endocardium** | **RV % fibrosis** | **RV collagen: myocyte ratio** |
| --- | --- | --- | --- | --- | --- | --- |
| RV capillary density mean | - | 0.83 (0.63, 0.92) | 0.8 (0.6, 0.9) | 0.79 (0.6, 0.91) | 0.09 (-0.2, 0.37) | 0.05 (-0.24, 0.31) |
| RV capillary density sub epicardium | <0.001 | - | 0.56 (0.24, 0.79) | 0.46 (0.11, 0.72) | 0.22 (-0.14, 0.53) | 0.2 (-0.16, 0.51) |
| RV capillary density mid layer | <0.001 | <0.001 | - | 0.49 (0.17, 0.77) | -0.02 (-0.35, 0.31) | -0.03 (-0.36, 0.31) |
| RV capillary density sub endocardium | <0.001 | 0.003 | 0.001 | - | -0.01 (-0.34, 0.34) | -0.04 (-0.38, 0.3) |
| RV % fibrosis | 0.53 | 0.18 | 0.88 | 0.93 | - | 0.98 (-1, 1) |
| RV collagen: myocyte ratio | 0.74 | 0.22 | 0.86 | 0.80 | <0.001 | - |

|  | **LV capillary density mean** | **LV capillary density sub epicardium** | **LV capillary density mid layer** | **LV capillary density sub endocardium** | **LV % fibrosis** | **LV collagen: myocyte ratio** |
| --- | --- | --- | --- | --- | --- | --- |
| LV capillary density mean | - | 0.84 (0.71, 0.92) | 0.89 (0.78, 0.94) | 0.56 (0.26, 0.76) | -0.16 (-0.41, 0.11) | -0.13 (-0.37, 0.14) |
| LV capillary density sub epicardium | <0.001 | - | 0.7 (0.51, 0.84) | 0.22 (-0.1, 0.51) | -0.05 (-0.38, 0.3) | 0.01 (-0.31, 0.34) |
| LV capillary density mid layer | <0.001 | <0.001 | - | 0.41 (0.08, 0.66) | -0.23 (-0.57, 0.13) | -0.14 (-0.5, 0.27) |
| LV capillary density sub endocardium | <0.001 | 0.18 | 0.010 | - | -0.31 (-0.58, 0.04) | -0.35 (-0.59, -0.02) |
| LV % fibrosis | 0.24 | 0.78 | 0.16 | 0.06 | - | 0.98 (-1, 0.99) |
| LV collagen: myocyte ratio | 0.36 | 0.93 | 0.41 | 0.030 | <0.001 | - |

**Table S6. “univariable” linear regression models adjusted for center. Coefficients of centers from each model were not shown.**

|  | **RV capillary density mean** | | | **RV capillary density sub epicardium** | | |
| --- | --- | --- | --- | --- | --- | --- |
| **Variable** | **Coefficient (95% CI)** | **p-value** | **N** | **Coefficient (95% CI)** | **p-value** | **N** |
| **Type of explant** - Combined | -33.50 (-170.21,103.20) | 0.63 | 55 | -89.19 (-279.00,100.62) | 0.36 | 42 |
| Isolated | -14.44 (-155.22,126.33) | 0.84 | 55 | -44.06 (-242.25,154.13) | 0.67 | 42 |
| **Age** | 2.54 (-1.07,6.15) | 0.17 | 55 | 3.84 (-1.76,9.44) | 0.19 | 42 |
| **Chronic kidney disease** | 71.56 (-28.64,171.75) | 0.17 | 55 | 30.36 (-103.67,164.40) | 0.66 | 42 |
| **Diabetes mellitus** | 41.59 (-74.71,157.89) | 0.49 | 55 | 101.67 (-50.82,254.17) | 0.20 | 42 |

|  | **RV capillary density mid layer** | | | **RV capillary density sub endocardium** | | |
| --- | --- | --- | --- | --- | --- | --- |
| **Variable** | **Coefficient (95% CI)** | **p-value** | **N** | **Coefficient (95% CI)** | **p-value** | **N** |
| **Type of explant** - Combined | -146.46 (-332.57,39.64) | 0.13 | 42 | -81.63 (-299.71,136.45) | 0.47 | 43 |
| Isolated | -102.32 (-296.65,92.00) | 0.31 | 42 | -10.53 (-239.14,218.09) | 0.93 | 43 |
| **Age** | 0.45 (-5.27,6.17) | 0.88 | 42 | -0.35 (-7.20,6.51) | 0.92 | 43 |
| **Chronic kidney disease** | 63.35 (-69.36,196.06) | 0.36 | 42 | 76.50 (-85.06,238.07) | 0.36 | 43 |
| **Diabetes mellitus** | 69.13 (-85.01,223.27) | 0.38 | 42 | 2.34 (-188.69,193.36) | 0.98 | 43 |

|  | **LV capillary density mean** | | | **LV capillary density sub epicardium** | | |
| --- | --- | --- | --- | --- | --- | --- |
| **Variable** | **Coefficient (95% CI)** | **p-value** | **N** | **Coefficient (95% CI)** | **p-value** | **N** |
| **Type of explant** - Combined | -88.66 (-230.54,53.22) | 0.23 | 55 | -137.56 (-360.48, 85.36) | 0.23 | 41 |
| Isolated | -95.41 (-241.51,50.69) | 0.21 | 55 | -9.44 (-235.95,217.07) | 0.94 | 41 |
| **Age** | -0.31 (-4.19,3.57) | 0.88 | 55 | 1.55 (-4.43,7.53) | 0.61 | 41 |
| **Chronic kidney disease** | -32.04 (-139.34,75.26) | 0.56 | 55 | -17.97 (-179.13,143.19) | 0.83 | 41 |
| **Diabetes mellitus** | -46.44 (-169.01,76.13) | 0.46 | 55 | 32.37 (-149.25,213.98) | 0.73 | 41 |

|  | **LV capillary density mid layer** | | | **LV capillary density sub endocardium** | | |
| --- | --- | --- | --- | --- | --- | --- |
| **Variable** | **Coefficient (95% CI)** | **p-value** | **N** | **Coefficient (95% CI)** | **p-value** | **N** |
| **Type of explant** - Combined | -107.90 (-288.45,72.65) | 0.25 | 41 | -160.48 (-345.33,24.36) | 0.10 | 41 |
| Isolated | -118.47 (-301.93,64.99) | 0.21 | 41 | -137.27 (-325.10,50.55) | 0.16 | 41 |
| **Age** | 0.48 (-4.32,5.28) | 0.85 | 41 | 2.68 (-2.24,7.61) | 0.29 | 41 |
| **Chronic kidney disease** | -43.71 (-171.97,84.55) | 0.51 | 41 | 56.21 (-76.82,189.25) | 0.41 | 41 |
| **Diabetes mellitus** | -23.33 (-168.69,122.03) | 0.75 | 41 | -107.86 (-255.28,39.55) | 0.16 | 41 |

**Table S7 Multivariable linear regression models adjusted for center**

|  | **RV capillary density mean**  **(N=55)** | | | **RV capillary density sub epicardium**  **(N=42)** | | |
| --- | --- | --- | --- | --- | --- | --- |
| **Variable** | **Coefficient (95% CI)** | **p-value** |  | **Coefficient (95% CI)** | **p-value** |  |
| **Institution** - Univ. Penn | -152.61 (-313.25,8.03) | 0.07 |  | -160.76 (-363.74, 42.21) | 0.13 |  |
| Univ. Utah | 59.08 (-66.73,184.89) | 0.36 |  | 173.49 (-2.85,349.83) | 0.06 |  |
| **Type of explant** - Combined | -42.72 (-190.06,104.63) | 0.57 |  | -85.52 (-288.62,117.57) | 0.41 |  |
| Isolated | -39.90 (-198.96,119.17) | 0.63 |  | -62.24 (-292.08,167.60) | 0.60 |  |
| **Age** | 1.51 (-2.65,5.67) | 0.48 |  | 2.57 (-3.60, 8.73) | 0.42 |  |
| **Chronic kidney disease** | 66.79 (-49.71,183.29) | 0.27 |  | 14.55 (-148.36,177.45) | 0.86 |  |
| **Diabetes mellitus** | 19.73 (-105.28,144.73) | 0.76 |  | 80.18 (-87.51,247.88) | 0.36 |  |

|  | **RV capillary density mid layer (N=42)** | | | **RV capillary density sub endocardium (N=43)** | | | |
| --- | --- | --- | --- | --- | --- | --- | --- |
| **Variable** | **Coefficient (95% CI)** | **p-value** |  | | **Coefficient (95% CI)** | **p-value** |  |
| **Institution** - Univ. Penn | -131.58 (-329.47, 66.32) | 0.20 |  | | -220.91 (-463.29, 21.46) | 0.08 |  |
| Univ. Utah | 45.18 (-126.75,217.11) | 0.61 |  | | -3.61 (-223.16,215.94) | 0.97 |  |
| **Type of explant** - Combined | -194.43 (-392.44,3.59) | 0.06 |  | | -130.86 (-372.53,110.81) | 0.30 |  |
| Isolated | -187.95 (-412.05, 36.14) | 0.11 |  | | -90.32 (-365.41,184.77) | 0.52 |  |
| **Age** | -1.63 (-7.65,4.38) | 0.60 |  | | -1.78 (-9.34,5.77) | 0.65 |  |
| **Chronic kidney disease** | 109.15 (-49.68,267.98) | 0.19 |  | | 110.65 ( -92.42,313.71) | 0.29 |  |
| **Diabetes mellitus** | 57.41 (-106.09,220.91) | 0.50 |  | | -16.57 (-225.27,192.13) | 0.88 |  |

|  | **LV capillary density mean**  **(N=55)** | | | **LV capillary density sub epicardium (N=41)** | | |
| --- | --- | --- | --- | --- | --- | --- |
| **Variable** | **Coefficient (95% CI)** | **p-value** |  | **Coefficient (95% CI)** | **p-value** |  |
| **Institution** - Univ. Penn | 70.49 (-100.87,241.86) | 0.42 |  | -101.92 (-346.48,142.64) | 0.42 |  |
| Univ. Utah | 181.24 (47.04,315.45) | 0.011 |  | 144.63 (-64.81,354.06) | 0.19 |  |
| **Type of explant** - Combined | -90.34 (-247.52, 66.85) | 0.27 |  | -119.94 (-366.27,126.39) | 0.35 |  |
| Isolated | -93.59 (-263.28, 76.09) | 0.29 |  | 21.37 (-242.78,285.53) | 0.87 |  |
| **Age** | -0.65 (-5.08,3.79) | 0.78 |  | 1.05 (-5.84,7.95) | 0.77 |  |
| **Chronic kidney disease** | 0.03 (-124.25,124.31) | >0.99 |  | -49.63 (-236.06,136.80) | 0.61 |  |
| **Diabetes mellitus** | -28.49 (-161.84,104.87) | 0.68 |  | 40.80 (-161.73,243.33) | 0.70 |  |

|  | **LV capillary density mid layer (N=41)** | | | **LV capillary density sub endocardium (N=41)** | | | |
| --- | --- | --- | --- | --- | --- | --- | --- |
| **Variable** | **Coefficient (95% CI)** | **p-value** |  | | **Coefficient (95% CI)** | **p-value** |  |
| **Institution** - Univ. Penn | -134.71 (-334.02, 64.60) | 0.19 |  | | 107.18 (-80.13,294.49) | 0.27 |  |
| Univ. Utah | 191.03 (20.34,361.71) | 0.035 |  | | 117.64 (-42.77,278.05) | 0.16 |  |
| **Type of explant** - Combined | -100.31 (-301.06,100.45) | 0.33 |  | | -156.38 (-345.05, 32.28) | 0.11 |  |
| Isolated | -107.30 (-322.58,107.98) | 0.34 |  | | -167.21 (-369.53, 35.11) | 0.11 |  |
| **Age** | 0.21 (-5.42,5.83) | 0.94 |  | | 2.86 (-2.42,8.15) | 0.30 |  |
| **Chronic kidney disease** | -15.97 (-167.91,135.97) | 0.84 |  | | 112.60 (-30.19,255.39) | 0.13 |  |
| **Diabetes mellitus** | -14.92 (-179.98,150.14) | 0.86 |  | | -157.48 (-312.60, -2.35) | 0.055 |  |

**Table S8. Multivariable linear regression for RV fibrosis and RV collagen: myocyte ratio adjusted for center**

|  | **RV % Fibrosis**  **N=51** |  | **RV collagen: myocyte ratio**  **N=51** |  |
| --- | --- | --- | --- | --- |
| **Variable** | **Coefficient (95% CI)** | **p-value** | **Coefficient (95% CI)** | **p-value** |
| **Institution** - Univ. Penn | -0.74 (-1.77,0.28) | 0.16 | -0.02 (-0.33,0.29) | 0.90 |
| Univ. Utah | -0.24 (-0.99,0.51) | 0.53 | 0.09 (-0.12,0.31) | 0.40 |
| **Type of explant** - Combined | -0.86 (-1.73,0.01) | 0.06 | -0.18 (-0.44,0.08) | 0.18 |
| Isolated | -0.81 (-1.78,0.16) | 0.11 | -0.12 (-0.41,0.17) | 0.42 |
| **Age** | -0.01 (-0.03,0.02) | 0.51 | -0.01 (-0.01,0.00) | 0.050 |
| **Gender** - Male | 0.36 (-0.28,1.00) | 0.28 | 0.11 (-0.09,0.30) | 0.29 |
| **Diabetes mellitus** | -0.33 (-0.99,0.33) | 0.33 | -0.09 (-0.28,0.10) | 0.37 |
| **Etiology of heart failure** - Congenital | 0.21 (-0.98,1.40) | 0.74 | 0.12 (-0.23,0.47) | 0.51 |
| Ischemic | 0.76 (-0.59,2.12) | 0.28 | 0.22 (-0.18,0.62) | 0.28 |
| Myocarditis | -0.27 (-2.01,1.47) | 0.76 | -0.09 (-0.61,0.42) | 0.72 |
| Other | 0.76 (-0.26,1.78) | 0.15 | 0.19 (-0.11,0.49) | 0.23 |
| Valvular | -0.40 (-2.58,1.78) | 0.72 | -0.06 (-0.71,0.58) | 0.85 |
| **RV diastolic pressure** | -0.02 (-0.09,0.06) | 0.65 | 0.01 (-0.01,0.03) | 0.39 |
| **RA pressure** | 0.02 (-0.05,0.09) | 0.54 | -0.01 (-0.03,0.01) | 0.49 |

Legend: Sensitivity analysis including center as a fixed effect. Model covariates identical to Table 8. No independent association was found between PH subtype and RV fibrosis after additional adjustment of institutes which account for variability between centers.
